# A robust model of Stimulus-Specific Adaptation validated on neuromorphic hardware

**DOI:** 10.1038/s41598-021-97217-3

**Published:** 2021-09-09

**Authors:** Natacha Vanattou-Saïfoudine, Chao Han, Renate Krause, Eleni Vasilaki, Wolfger von der Behrens, Giacomo Indiveri

**Affiliations:** 1 Institute of Neuroinformatics, University of Zurich and ETH Zurich, Zurich, Switzerland; 2 Department of Computer Science, University of Sheffield, Sheffield, UK

**Keywords:** Neuroscience, Mathematics and computing

## Abstract

Stimulus-Specific Adaptation (SSA) to repetitive stimulation is a phenomenon that has been observed across many different species and in several brain sensory areas. It has been proposed as a computational mechanism, responsible for separating behaviorally relevant information from the continuous stream of sensory information. Although SSA can be induced and measured reliably in a wide variety of conditions, the network details and intracellular mechanisms giving rise to SSA still remain unclear. Recent computational studies proposed that SSA could be associated with a fast and synchronous neuronal firing phenomenon called Population Spikes (PS). Here, we test this hypothesis using a mean-field rate model and corroborate it using a neuromorphic hardware. As the neuromorphic circuits used in this study operate in real-time with biologically realistic time constants, they can reproduce the same dynamics observed in biological systems, together with the exploration of different connectivity schemes, with complete control of the system parameter settings. Besides, the hardware permits the iteration of multiple experiments over many trials, for extended amounts of time and without losing the networks and individual neural processes being studied. Following this “neuromorphic engineering” approach, we therefore study the PS hypothesis in a biophysically inspired recurrent networks of spiking neurons and evaluate the role of different linear and non-linear dynamic computational primitives such as spike-frequency adaptation or short-term depression (STD). We compare both the theoretical mean-field model of SSA and PS to previously obtained experimental results in the area of novelty detection and observe its behavior on its neuromorphic physical equivalent model. We show how the approach proposed can be extended to other computational neuroscience modelling efforts for understanding high-level phenomena in mechanistic models.

## Introduction

The auditory environment is composed of a significant amount of complex sounds from different sources that need to be organized by the nervous system to efficiently achieve stimulus processing. To organize this composite environment, the auditory system uses the surrounding sounds and their associated characteristics (e.g. the frequency of presentation of a stimulus^1^) and determines if these events are behaviorally important, considering that rare events could potentially have a higher relevance. In human electroencephalogram (EEG) recordings and in response to auditory stimuli, a correlate of change has been identified in the form of an additional negative wave called mismatch negativity (MMN). This wave is visible in the EEG after stimulus onset in response to a rarely occurring stimulus (termed as “deviant”) embedded randomly in a sequence of repeated standard tone stimulus^2–5^. Although MMN has been recognized as a robust neuropsychiatric and neurocognitive biomarker for many disorders, a detailed understanding of this phenomenon at the cellular and neuronal level remains to be clarified. A pertinent neural mechanism that has been proposed for this phenomenon is Stimulus-Specific Adaptation (SSA)^6,7^. SSA is the decrease in neuronal spiking response to a frequently presented stimulus that is not occurring to a rarely appearing event. Despite prevalent SSA distribution in the auditory system^6,8–11^, the precise cellular mechanism underlying this phenomenon remains largely unknown. Supported by computational modelling studies, the hypothesis of a depressed feed-forward thalamocortical input to the primary auditory cortex (A1) could explain SSA^12–14^. However, this hypothesis requires further investigation as the prediction suggested by these models does not fully concur with the experimental data^15^. For instance, these models could not successfully predict the correct size of the response to a rare stimulus in the experiments that control for rarity. Also, these models under-predicted the response to the deviants when compared to all other oddball conditions^16^. These results led to the belief that additional mechanisms were required in the genesis of SSA in the auditory cortex. Thus, in 2017, Yarden et al.^15^ proposed a novel mechanism for SSA involving both a recurrent network and local synaptic depression. Based on the previously mentioned neural network architecture, the work presented here provides further support for a recurrent network hypothesis in the generation of SSA, using for the first time both a mean-field model and a neuromorphic implementation. In addition, the necessity for the development of SSA of a synchronous firing event called Populations Spikes through the connection recurrency of the network is also shown in this study. Similar to earlier studies that combine mean-field models and microscopic simulations^17^, we explored the properties of the neural network with a minimal mean-field rate model and implemented it on a mixed-signal analog-digital neuromorphic hardware circuit.

The hardware used in the present study is the Dynamic Neuromorphic Asynchronous Processor (DYNAP-SE) originally presented by Moradi et al.^18^. The neuromorphic approach aims to understand and characterize the complex processes of neuronal circuits via the use of silicon neurons, a crucial element in building biologically similar neural networks^19,20^. This methodology allows real-time processing and ultra-low power consumption moving forward the development of autonomous systems^21^. The neuromorphic substrate can directly emulate properties of real neurons and synapses in real-time, that are not always accounted for in the software simulations, such as noise, component variability, limited precision and other phenomena present in biology. For instance, most of the computational neuroscience simulations of neural networks neither restrict the resolution of the synaptic weights to a few bits nor add variability to state variables to model diversity in real neurons and synapses. Moreover, to the best of our knowledge, and although it is possible in principle to study parameters variability in learning performance using software simulation, this has only been demonstrated recently in practice^22^. The neuromorphic recurrent neural network was configured to produce SSA and allow to further test the computational properties of this phenomenon. The models presented in this work were inspired by the neuroanatomy of A1 and the results were compared to previously published experimental and software modelling data. The neuromorphic model used a biologically plausible substrate and allowed us to explore the computational role of population spikes (PS), i.e., a large group of neurons firing briefly and synchronously, whose existence has been proposed by numerous studies conducted in the auditory system and particularly in A1. Intracellular recordings studies^23^ conducted along the auditory pathway, in auditory stations such as the medial geniculate body, the inferior colliculus, and in the primary auditory cortex, pointed out the existence of the PS phenomenon using low-level tones. Similarly, using a low-firing rate environment, *in vivo* whole-patch clamp recordings demonstrated that a highly organized presynaptic inputs activity was able to induce “bumps” in postsynaptic voltage, in other words, was able to activate strongly and shortly a large group of neurons synchronously^24^.

Our model supports the hypothesis proposed to the Yarden et al.’s hypothesis^15^ that PS are essential in the development of SSA. This intrinsic phenomenon, associated with the properties of the model, propagates laterally upon a rare (i.e. deviant) stimulus presentation. We show the occurrence of SSA in a real-time neural processing hardware with the involvement of PS and thereby provide further support for the previously proposed recurrent model of SSA.

## Results

The present work recreated a simplified version of the network presented in^15^ while using a mean-field rate model and an implementation on a neuromorphic hardware. The latter represents a more realistic platform which aims at reproducing the dynamics and computational functions present in biological brains. The reason we adopted the mean-field model is that we can treat the simplified network analytically under certain assumptions and derive the necessary conditions to show SSA and its properties. Consequently, this provided insights for tuning the neuromorphic model. In terms of population activities, the mean-field rate model is theoretically equivalent to a homogeneous group of leaky integrate-and-fire neurons^25^. The network presented holds special features to observe SSA such as recurrent connectivity, PS propagation and an STD-like mechanism.

### Mean-field model

**Figure 1:**
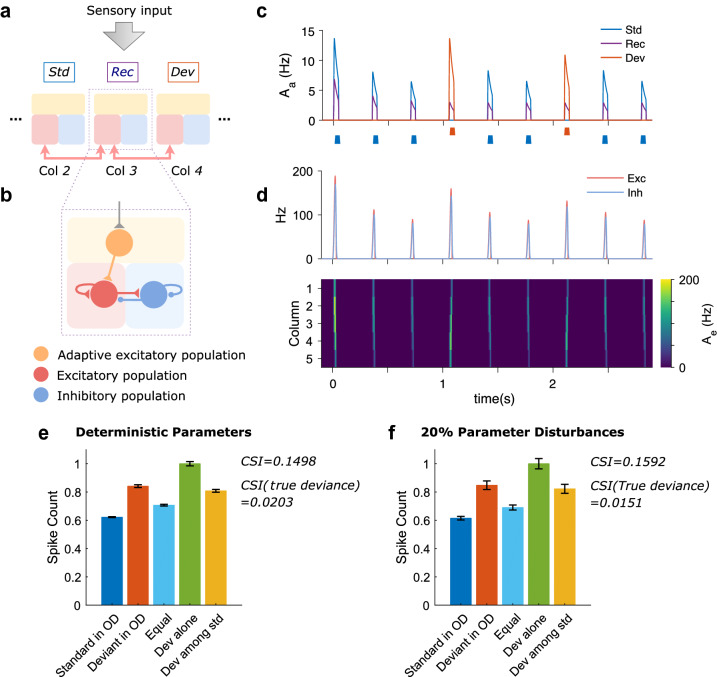
Oddball paradigms and SSA response of the network using a mean-field model. **(a)** The network model is composed of five cortical columns that are arranged along the frequency axis by their preferred frequencies (only the central three columns are shown here). Each column is constructed in three blocks: the adaptive layer (light yellow) receives thalamocortical sensory inputs and sends its feedforwards outputs to the excitatory (light red) population that is coupled with the inhibitory (light blue) population. The excitatory group is also connected to its nearest lateral excitatory counterparts, illustrated by arrows. During a “traditional” oddball paradigm, two different sensory stimuli are applied respectively to two symmetrical columns in our network: the standard (blue box) stimulates the left column at the probability of appearance of 0.75, while the deviant (red box) stimulates the right column with a probability of 0.25. The recording column in midway is the one from which the outgoing spikes are recorded (purple box). **(b)** To be detailed, within every column, the feedforward adaptive population excites a recurrent excitatory population that is regulated by another recurrent inhibitory population. The excitatory group is also activated by excitatory populations in its bilateral columns. **(c)** The adaptive population activities $A_a$ in the standard column (blue) are often weaker than the deviant (red), yet either the standard or deviant stimuli always trigger the same extent of activities in the recording column (purple) after the first several trials of stimulation. The stimuli sequence is shown under the plot. **(d)** Excitatory (pale red) and inhibitory (pale blue) populations in the recording column (column 3) often respond stronger to the deviant compared to the standard stimulation (top) because excitatory population activities $A_e$ in column 4 initiated by the deviant usually propagate more significantly into recording column midway than by the standard-triggered activities in column 2 (bottom). The time axis is vertically aligned for (**c**,**d**). **(e)** To investigate the deviant response characteristic, 5 control oddball (OD) protocols were used: “Standard in OD” in which a stimulus is presented to column 4 as a standard with a 75% probability of appearance (blue), “Deviant in OD” in which the same stimulus applied to column 4 was used as a deviant with a 25% probability of appearance (red), the “Equal” paradigm where two stimuli are presented 50% of the time each to column 2 and 4 respectively (cyan), “Dev alone” (green) where the stimulus is presented to column 4 alone as a deviant against silence and the “Dev among Std” (yellow) in which four different stimuli are presented 25% of the time each to column 1, 2, 4 and 5, respectively. The average responses in the recording column (spike count) to 5 different protocols are presented with the bar height representing the mean spike count with the standard error of the mean. The spike counts in each protocol are normalized to the Deviant alone condition. The data are averaged over responses of recording column to column 4 stimulation in each protocol consisting of 800 stimuli in total. In all simulation protocols, network parameters are chosen from Table 2, input amplitude *A* = 15 spikes/s, stimulus duration = 50 ms and inter-stimulus interval (ISI) = 300 ms (stimulus offset to onset). **(f)** The same setup was employed as **(e)**, except that 20% real-time parameter disturbances were introduced to parameters in Table 2, excluding the number of columns *M*, the width of tuning curve $\lambda$ and the membrane time constants $\tau , \tau _e, \tau _i$ that were taken from the literature^15^.

As illustrated in Fig. 1a, the primary auditory cortex is modelled as coupled cortical columns, each of which consists of two cascaded layers: the adaptive layer where neurons demonstrate spike-frequency adaptation and the recurrent excitatory-inhibitory layer. Columns are arranged along the frequency axis by their preferred frequency. Adaptive population in each cortical column is evoked by feedforward thalamocortical inputs dependent on the frequency interval between the preferred frequency of the column and the stimulated frequency. Typically, the farther the stimulated frequency is from the preferred frequency of a column, the weaker the thalamocortical input the column will receive (see the “Methods” section for the specific tuning curve of the column’s thalamic input used). The adaptive population subsequently activates the recurrent excitatory group regulated by the recurrent inhibitory group (Fig. 1b). The excitatory population also receives direct input from other excitatory populations in its adjacent columns (Fig. 1a).

SSA emerges in the network in response to a “traditional” oddball paradigm, as shown in Fig. 1d, top. This “traditional” oddball paradigm (OD) consists of two stimuli, standard and deviant, which differ in at least one stimulus feature (such as frequency or amplitude) activating different populations of neurons. In this study, the standard stimulus is presented with a higher probability than the deviant stimulus. In order to control for potential asymmetries in processing the two stimuli (e.g., being more sensitive to one stimulus frequency than the other) a second oddball paradigm is presented where the occurrence probabilities of the two stimuli are switched and the stimulus that was earlier the standard is now the deviant. Such a flip-flop paradigm allows now to compare the responses to an identical stimulus, when presented as standard and as deviant. Due to the higher occurrence probability of the standard stimulus, it usually induces much more adaptation in the standard column population than the deviant does in its column (Fig. 1c). The excitatory population in the deviant column receives lesser adapted inputs from the adaptive layer than the standard column, and thus, generates more robust responses. Consequently, more substantial activities are propagated into the middle column by the deviant column than the standard (Fig. 1d), leading to the generation of SSA.

It should be noted that the presence of PS in the auditory cortex has been widely observed in experiments^26–28^. The synchronized activities enable their lateral propagation across multiple cortical columns, leading to the generation of SSA. From a modelling point of view, prolonged activation could also implement the lateral propagation of activities by increasing the excitatory population’s firing rate and then spreading to its neighbouring populations via inter-column connections, similarly as population spikes do. However, to match the population bursts recorded in A1^26–28^, we model response to sensory stimulation as a transient synchronization in population activities (PS).

Besides the “traditional” OD protocol, three more protocols were tested on our network (“Equal”, “Dev alone”, and “Dev among std”) which we also employed to test true deviant detection (TDD) on the model (Fig. 1e). The trends, across the different protocols, are in line with the experimental findings in rat primary auditory cortex^14^. For the “traditional” and equal oddballs, when the deviant column was stimulated, its excitatory populations received less adapted inputs due to the rarity of the presentation of this stimulus. Consequently, more robust activities were propagated into the recording middle column, leading to the strongest response observed in the “Deviant in OD” protocol, followed by the “Equal” response and finally, the lowest response of the network with the “Standard in OD”. Besides, the Deviant alone protocol (“Dev alone”) evoked the largest response among all conditions as there was no cross-frequency adaptation caused by other stimuli. At last, the condition for the network demonstrating TDD is given in Eq. (17) (see “Methods” for details). Both positive CSI values (CSI = 0.1495, CSI (true deviance) = 0.0203) indicate the existence of SSA and TDD in the simulated network model.

To test the robustness of the mean-field model, except for the network structure parameters, the adaptation and the membrane time constants that are based on the literature^15^, we simultaneously varied the rest of the free network parameters in Table 2 by adding 20% online perturbations of their optimal values, leading to the fluctuation of the parameters uniformly bound between 1-20% and 1+20% of their optimal values. We then averaged the responses of column 3 to the tone frequency of 4 Hz within each protocol as we did for the deterministic parameter condition. As a result, one can see that the parameter perturbation only increase the response variations and has a negligible effect on SSA according to the CSI values calculated from the mean responses (CSI = 0.1592, CSI (true deviance) = 0.0151) (Fig. 1f). This demonstrates the robustness of our model against online parameter disturbances. In addition, we also did the sensitivity analysis of the free network parameters by varying each of them individually (with all the others fixed in their optimal values) and explored the operating range of the parameters where SSA and TDD are both valid, based on positive CSI metrics. From parameter ranges reported in Table 2, we can see that the SSA and TDD of our model are relatively sensitive to the excitatory connection weights and then the inhibitory connection weights, but much less susceptible to the adaptation connection weight and adaptation scaling factor. The results meet our expectation since one critical element of SSA in our model is the extent of the spread of excitatory activities across columns that is mainly restricted by the strength of intra-column recurrent excitatory and inter-column connections. Another key element of SSA is the recovery time from the adaptation, parameter previously reported in the literature^29^.

### Neuromorphic model

#### Generation of populations Spikes and simulated-STD

**Figure 2:**
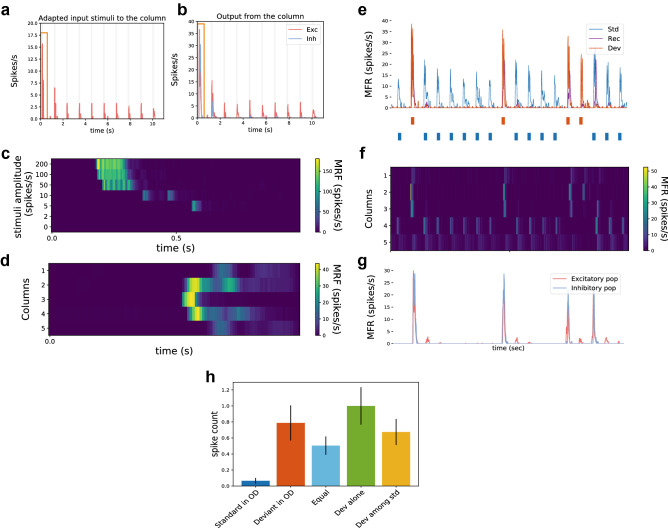
Neuromorphic implementation of SSA. Ten stimulations have been applied to the middle column to analyze our simulated-STD and the behaviour of a column in the network upon multiple stimulations. **(a)** represents the input coming to the column and **(b)** correspond to the output from the column. Synaptic depression has been previously shown to be key in the generation of PS and our model of spike frequency adaptation does simulate synaptic depression by the use of adapted inputs generated by the adaptive layer **(a)**. This results in an increase of the transient MRF upon presentation of the first stimulus (orange rectangle) which does not occur for the next presentations **(b)**. A PS appears upon the arrival of a stimulus and is represented by an increased MRF of the excitatory neuronal population in the column (red graph). The inhibitory population’s MRF (blue graph) increases only when a PS is generated and decreases through the course of the stimulation. Stimulus presentations (shown in gray) are of 100ms long followed by a pause of 900ms in between. **(c)** and **(d)** represents respectively the PS property and development in our network neuromorphic implementation. The time course of PS generation is depending on stimuli of varying amplitude and confirms the threshold for PS generation **(c)**. Below an input stimulus of 5 spikes/sec, no PS were generated. The color scale represents the response strength. A PS appears upon presentation of input spikes and propagates through the network. The columns’ population activity **(d)** and the oscilloscope traces (see Supplementary data S2) demonstrate the propagation of spikes from the column where spikes were sent to the two adjacent columns. Following is the study of the dynamics of the network population in response to an oddball paradigm. **(e)** The firing rate of the network population of three columns (Deviant column (Dev), Recording column (Rec) and, Standard column (Std)) during an oddball paradigm show the appearance of SSA. PS were usually observed upon presentation of the deviant (stimuli presentation shown in blue (standard) and red (deviant) under the plot). **(f)** Upon presentation of the deviant, PS are propagating to one column from each side while the standard does not generate a PS, due to the simulated-STD. **(g)** The behaviour of both populations (excitatory and inhibitory) in the recording column has been studied and the activation of the inhibitory population occurs only while the deviant is presented. **(h)** The averaged responses of the recording column (spike count) following the different control OD protocols (as in Fig. 1e) were analysed, with the total spike counts normalized to the Deviant Alone condition. Each protocol consisted of 100 stimuli presentations, repeated 10 times, with an ISI = 1 second and the bar height represents the mean spike count with standard error of the mean. Similar results were obtained between the mean-field rate modelling and the neuromorphic implementation showing a tendency towards true deviance detection.

PS are occurrences in which numerous neurons are mobilized to fire at least one spike in a very short period, which is a major property of recurrent networks with synaptic depression^30–32^. Our model responded by generating PS upon arrival of a stimulus demonstrated by the increase of the transient mean firing rate (MFR) in a column of our network (see Fig. 2a,b). In this figure, inputs and outputs from one column upon ten stimulations of 100 ms each with an interstimulus interval (ISI) of 1 second (onset-to-onset) are presented. Figure 2a represents the output signal coming from the adaptive layer and demonstrates that our model can send adapted input to the network. This further generates a simulated-STD shown by a progressive decrease of the MFR upon repeated stimuli presentation. The observed fast adaptation mechanism is consistent with equivalent mechanisms that have been demonstrated in *in vivo* recordings^6,33^. Figure 2b shows the outputs coming from the column after reception of the adapted inputs and the behaviour of both excitatory (red curve) and inhibitory populations (blue curve) in the column through the course of the stimulation. The highlighted section in Fig. 2a,b (orange rectangle) indicated the apparition of PS upon presentation of the first stimulus which does not appear when further stimuli are applied. The column’s response following a PS is weaker due to the simulated-STD generated by the incoming adapted inputs to the column which, as a result, makes the recurrent excitation weaker. This prevents the formation of another PS by keeping the neurons adapted upon the arrival of a second stimulus. This will only be possible if there is synaptic availability and is a regenerative process. PS are evoked above threshold (see Fig. 2c), with a higher peak firing rate associated with a stronger stimulus input amplitude. In this figure, the color scale represents the neuronal response strength of the middle column upon presentation of different stimuli (ranging from 2 to 200 spikes/s).

#### Populations Spikes behaviour through the network

Once initiated, the model implemented on neuromorphic hardware demonstrated PS propagation from one column to the next (Fig. 2d). Here, the columns’ neuronal population activity propagation (Fig. 2d) and the oscilloscope membrane potential traces from three columns and one adaptive layer (Supplementary data S2) showed the propagation of spikes from the column where the PS were initiated, to the adjacent columns. We could then observe that a couple of milliseconds were required for the PS to reach the adjacent columns. This delay in spike propagation (i.e. spike latency) is in corroboration with observation in similar theoretical model^18^ and *in vivo* experiments^34^. As our model contains only five columns, a PS propagates through the entire network. This observation is reinforced by the fact that evoked PS usually propagates on each side of the column where it is initiated into a couple of columns^15^.

#### Implementation of SSA

The network, implemented on the neuromorphic hardware DYNAP-SE, shows a very persistent (Supplementary data S3) and strong SSA (Fig. 2e–g) across trials.

Figure 2e–g shows the neuromorphic implementation of our network and how it responded during one “traditional” OD paradigm (100 stimuli). The columns responses to the OD (Fig. 2e), the PS propagation through the network (Fig. 2f), and the MRF response of the excitatory and inhibitory population in the middle column (Fig. 2g) shows the occurrence of SSA in our network. The stimuli presentation shown under Fig. 2e (blue for the standard and red for the deviant stimuli presentation) applies to Fig. 2e–g data.

Figure 2e represents the MRF response of three columns (“standard” column, “deviant” column, and in midway between the two, the “recording” column) over the course of an OD. SSA occurs, in our neuromorphic model, when the deviant is presented (generating an average of 30 spikes/s response) while the standard is mostly not able to produce any PS due to the adapted state of its column (generating an average of 5 spikes/s response). The response to SSA is strong and consistent across trials with a CSI = 0.88. The network consistent behavior (Supplementary data S3) is visible through its consistent response when the deviant is presented or when the standard is presented for the first time (considered then technically as a novel stimulus). It is worth noting that when deviant stimuli are presented relatively close in time (Supplementary data S3, fifth and seventh deviant presentations), the network failed to produce SSA on the second presentation due to the increase of presentation probability similar to in vivo SSA studies^35^. Figure 2f represents the PS propagation into the whole network with the deviant stimulus applied to the “deviant” column (column 2) and the standard stimulus applied to the “standard” column (column 4). The data shows that the “standard” column, due to its multiple stimulations, is adapted. Hence, it is not able to generate a PS while the deviant presentation usually creates a PS that propagates into the “recording” column (column 3). This phenomenon also depends on the interstimulus interval: when two deviant stimuli were occurring one after the other, the second presentation was not able to generate a PS while the following standard presentation could (see Supplementary data S3, third and fourth deviant presentations). Figure 2g represents the excitatory and inhibitory population’s responses to an OD in the “recording” column due to the propagated PS in this column. In our model and at the beginning of the oddball paradigm, neurons needed to receive at least two to three consecutive stimuli to be fully adapted. This behaviour of our neuromorphic substrate explains the different firing patterns observed here compared to synaptic depression studies^10,15^ in which the second stimuli presentation will already induce an extremely lower synaptic response due to the depleted synaptic resources (see Supplementary data S3). To better assess the potential of this network, further OD paradigms were carried out.

#### Different OD paradigms and behaviour of our network

The “traditional” OD paradigm (Fig. 2a) does not allow to distinguish between SSA and true deviance detection as it involved the recognition of disruptions in a regular and predicted sequence. Therefore, a series of “control” OD paradigms (Fig. 2h) was tested both in the mean-field rate model and on the neuromorphic hardware. The control OD paradigms used were as follows: the Deviant alone (“Dev alone”) protocol, similar to the “traditional” OD paradigm with a 25% of appearance for the deviant but with the difference that the standard was omitted and the “Equal” protocol in which both stimuli were presented 50% of the time to make sure that there is no preferential behaviour of the network. Besides, to see if true deviance detection was possible, we used the Deviant among Standards protocol (“Dev among std”) which involved the presentation of the standard and deviant embedded in a sequence containing several other stimuli, all presented with a 25% probability of appearance. Thus, the probability of appearance of each stimulus, in this protocol, was equal to the one of the deviant stimulus. The deviant response in this protocol was then compared to the deviant response obtained from the “Deviant in OD” paradigm. In this protocol, there is no regularity established as the stimuli presentation is random. If the responses to the deviant from both the “traditional” and the “Dev among std” OD were found similar, then the response to the deviant could only be justified by SSA. Consequently, if the response to the deviant in the “traditional” OD is larger than the same deviant response from the “Dev among std” protocol then we would have confirmation of true deviance detection. In both mean-field modelling and neuromorphic implementation, the results tend to display true deviance detection with a CSI (true deviance) for the neuromorphic implementation of 0.1 and a CSI (true deviance) of 0.0203 for the mean-field rate.

## Discussion

The central goal of this study was to investigate the fundamental phenomenon of change detection. To this end, we adopted an approach whereby on one hand, we reproduced SSA using a simplified mean-field rate model of a previously reported neuronal network of SSA. On the other hand, we emulated, with real-time physical models of neurons and synapses, the properties of this recurrent network. The latter was accomplished with a hardware substrate that has properties and limitations similar to those measured from real neural processing systems. To realize these objectives, we used a mean-field model and a neuromorphic hardware, employing a spike-frequency-based adaptation approach and an anatomically plausible recurrent connectivity scheme. Both models in this study presented comparable results in terms of network activity, response to the OD paradigms and deviance detection. Their features also share similarities based on the minimal requirements used to observe SSA such as, the limited number of columns, recurrent connectivity and STD-like feature (spike-frequency adaptation). We show how these mechanisms were sufficient to give rise to SSA which is consistent with previous findings^36^ although SSA is a phenomenon emerging from several homeostatic processes^37^. The network behaviour remained consistent across trials, exhibiting a strong SSA as well as a reliable strong response from the network from each initial standard presentation and increasing its MRF response only when the deviant was subsequently presented.

The strong increase in MFR with the deviant presentation is also observed in biological neurons^37^. This result, together with the standard-induced adapted network response, suggest that SSA could be associated to the brain computational capacity in reducing input redundancy, consistent with biological findings^14,15,38^. The architecture implemented on both models solely used five cortical columns which impacted the study of true deviance detection. Although both models showed a tendency towards deviance detection, the results were not strongly conclusive due to the limited columnal architecture and thus, the number of “standard” stimuli used in the “Dev among std” protocol. Previous neuronal modellings of A1 have used in their implementation up to 21 interconnected recurrent cortical columns^15,30,39–41^. These architectures enabled the analysis of true deviance detection by using several “Dev among std” protocols to analyse the effects of the deviant in a regular background compared to an irregular one. Thus, more cortical columns for the network and a diverse “Dev among std” protocol will be used, in future studies, to determine true deviance detection in both mean-field model and its neuromorphic implementation.

The spiking neural network architecture is not only designed following the principles of biological nervous systems but also, processes information using energy-efficient, asynchronous and event-driven methods^19,42^. The neuromorphic hardware comes with constraints (from limited resolution, variability in the parameters and the state variables to device mismatch) that need to be accounted when creating a neuronal model. The real-time execution and built-in parameters of the neuromorphic hardware are useful in exploring SSA responses from the network. The hardware also allowed us to study PS in the presence of noise which is often disregarded in software models as it is seen as a hindrance for reliable information extraction. These unique features of the biophysically inspired neuromorphic hardware differentiate our approach^43,44^ from the conventional mathematical models used in the study of SSA. Furthermore, our method allows an intuitive assessment of the effect of different network configurations and parameter settings. The software as well as the hardware model presented here confirm that PS play an important role in SSA. Additionally, the hardware model highlights the importance of PS not only for SSA itself but also for stable signal propagation in a noisy substrate similar to the brain. This provides further support for the PS hypothesis and shows the benefit of using a more realistic modelling tool in neuroscience such as the neuromorphic hardware.

This work demonstrates that a network with parameters such as recurrent dynamic and spike-frequency adaptation is capable of producing SSA. Besides, this implementation allows the generation of PS which appears to be a pertinent mechanism for SSA as it is producing a particularly sharp neuronal response that appears generally when a deviant is presented and for a very short period. Our model is a simplified model in which for instance feedforward inhibition or cell types are not included but the simplification allowed us to focus on a minimum requirement of basic elements and demonstrates that they were sufficient to allow cortical SSA. The model still has some important elements of the organization of the rodent auditory cortex such as its columnal organization which simulates the smooth decay of connection strengths in the cortex^45^.

The majority of the SSA modelling studies have been based on the depression of the feedforward synapses^12,14,46^. As in the work of^15^, the SSA mechanistic underlined here differs from the previously reported mechanism by highlighting the importance of a recurrent network in the model studied. Besides Yarden and Nelken^15^, several studies have recently used computational models of SSA in the auditory cortex^36,47,48^. Park and Geffen^48^ in 2020 modelled the auditory circuitry with explicit anatomical connections, including multiple types of neurons (somatostatin- and parvalbumin- positive inhibitory interneurons and excitatory neurons). Such a detailed circuit model provided an excellent test setting for probing the consequences of circuit perturbations (e.g. silencing targeted types of neurons). This circuitry layed out an interesting comparison for our implementation. While our model did not go to this level of circuit details, it still reproduces qualitatively the main responses to the different stimulation paradigms and deviant configurations. Kudela et al.^36^ in 2018 used a multicompartmental cell model to investigate if neural fatigue or neural sharpening was involved in the mechanism behind cortical adaptation. Depending on the exact parameter settings, the authors could either achieve neuronal adaptation through a fatigue mechanism with synaptic depression or, through neural sharpening with the introduction of lateral inhibition. These adaptation effects were modelled not only on the level of single-neurons but also, for evoked cortical response. These results were particularly interesting for their comparison with human data and may facilitate a benchmarking of our neuromorphic model for comparative analysis with human data in the future. May and Westö et al.^47^ in 2015 use a Wilson Cowan Cortical Model^49^ with serial core-belt-parabelt structure of the auditory cortex. This structure, together with activity-dependent depression of excitatory corticocortical connections, was sufficient to generate SSA and future studies on the neuromorphic hardware will investigate such type of auditory cortex associated architecture.

True deviance takes into account not only the important neuronal response to the deviant but the identity of the other stimuli too. Thus, future steps will include the investigation of the network capacity to display true deviance sensitivity by using control protocols similar to the ones used in in vivo experiments^14,46^. The high capacity of the brain to compute external stimuli in an effective and fast manner leads to the aspiration of creating autonomous systems, capable of adapting and interact with their environment^50–52^. Yet, performing in real-time efficient novelty detection has been proven to be challenging due to the complexity of the environment and the need for accuracy^53^. Therefore, multicore platforms^43,53,54^ have been developed to permit high sensitivity and specificity in signal processing and detection. Besides, neuromorphic computing platforms offer a brain-like environment (such as the maintenance of noise in recordings) allowing the creation and study of robust networks with the possibility of modulating parameters^55^.

## Conclusions

The simplified version of the neural network presented in this study focuses on the role of intrinsic dynamics of recurrent cortical circuits and its implementation on low-powered neuromorphic hardware in SSA analysis. Conversely, this network does not take into account higher features such as the complex transformation of auditory inputs that occurred in subcortical regions. Future work will be aimed at building an even more complex network to capture the entire auditory organization and its function in novelty detection. The next generation of DYNAP-SE will also allow us to use new built-in features such as on-chip plasticity and synaptic depression, important in novelty detection.

## Methods

### Network architecture

**Figure 3:**
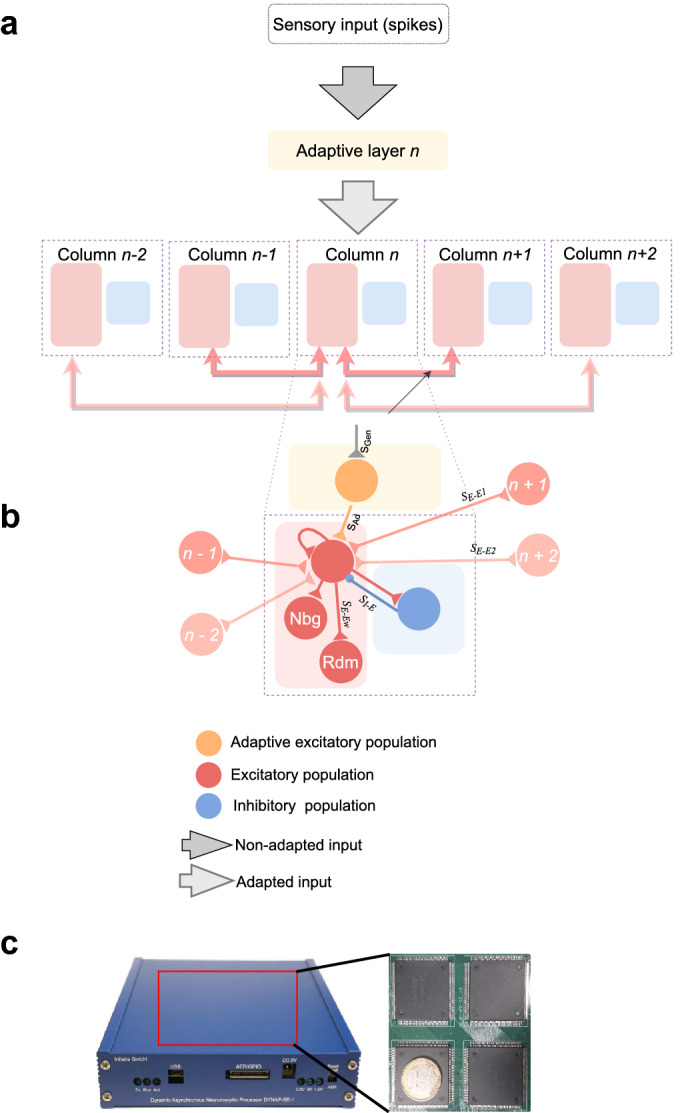
Network architecture (population and single neuron connectivity) and neuromorphic hardware used in this study. A network constituted of five cortical columns has been implemented using a recurrent neural network to represent the primary auditory cortex A1. **(a)** Arrows illustrate the connectivity of one column and the shades of the arrows the strength of its connections to the other columns (strength decreasing according to their distance to the column receiving the sensory input (S$_{E-E1}$ and S$_{E-E2}$). Spikes from the spikes generator are received by the adaptive layer (S$_{Gen}$). **(b)** Each column contains both excitatory (E) neurons and inhibitory (I) neurons with a ratio of 4/1 and receives adapted input spikes (S$_{Ad}$) from the adaptation layer which in return activates the whole column through the intracolumnal recurrent connections (S$_{E-EW}$) with random (Rdm) and neighbouring (Nbg) neurons. **(c)** The Dynamic Neuromorphic Asynchronous Processor Scalable (DYNAP-SE) board is composed by four chips comprising each four cores and each of the core possess 256 adaptive exponential integrate-and-fire neurons. The figure represents the external appearance of the neuromorphic processor (blue box) and the printed circuit board of the four chips. Neurons and synapses parameters can be programmed via an on-chip bias generator.

The architecture implemented on the neuromorphic hardware and used in the mean-field model is closely related to the one proposed in Yarden et al.^15^ of A1. In this study, we used a network comprising five interconnected cortical columns (Fig. 3a) each containing intracolumnal recurrent connections (Fig. 3b). These columns were distributed over five different cores of the DYNAP-SE to have more control over the parameters, as the biases of the DYNAP-SE are core-dependent. The parameters of the model were not changed during the experiments, as the model was behaving as expected without requiring extra ad-hoc parameter tuning. As the neuromorphic hardware is temperature-sensitive, all experiments were carried under fixed standard laboratory conditions in which the experiments were very reproducible from trial to trial. Table 1 presents the synaptic parameters of the middle column 3 consisting of 100 excitatory neurons and 23 inhibitory neurons. By keeping in mind that each neuron on the DYNAP-SE has only a fan-in of 64 and to obtain full recurrent connectivity, each excitatory neuron received input from its eight nearest neighbors, from eight neurons taken randomly within the column and from the 23 inhibitory neurons. All neurons also received input from the excitatory population of the nearest- and second-nearest neighboring columns, with connection strength that decreased according to intercolumnal distance (i.e. respectively eight excitatory neurons from column 4 and four excitatory neurons from column 5 projected to the excitatory population of column 3).

SSA is known to be generated through a combination of membrane excitability changes and short-term synaptic depression (STD)^56–58^. As the neuromorphic hardware used in this study does not possess STD, we employed a spike-frequency adaptation mechanism to simulate synaptic STD (Fig. 3a) through the introduction of an adaptive input layer. Spike-frequency adaptation is a phenomenon in which neurons show a reduction in their firing frequency of the spike response following an initial step increase^59^. In the present neuromorphic study, we will refer to this function of spike-frequency adaptation as “simulated-STD”. Furthermore, it is worth noting that the adaptation feature used in our network is an interesting tool to investigate SSA as past research has demonstrated that the cortical adaptation recovery time scale to tones was consistent with the cortical synapse recovery scale in synaptic depression^6,60,61^.

**Table 1:** Synapse parameters of column 3 of the network implemented on neuromorphic hardware.

Connections	Name	Connections strength	Polarity	Time constant ${\varvec{\tau }}$
Input spikes to adaptive layer	$S_{gen}$	[7 150]	exc	[2 48]
Input adaptive spikes to column	$S_{Ad}$	[ 7 200]	exc	[1 230]
Input excitatory neurons within the column	$S_{E-Ew}$	[3 230]	exc	[2 40]
Input spikes to excitatory neurons to column 4	$S_{E-E1}$	[2 127]	exc	[2 44]
Input spikes to excitatory neurons to column 5	$S_{E-E2}$	[2 40]	exc	[4 215]
Input spikes inhibitory neurons to Column	$S_{I-E}$	[4 130]	inh	[2 40]
Input spikes excitatory to inhibitory	$S_{E-I}$	[3 230]	exc	[2 40]

A connection between two neurons (Fig. 3b) is characterised by the positive (excitatory, exc) or negative (inhibitory, inh) current and the time constant. These parameters’ values [coarse and fine] are converted into currents using an 11-bits DAC (digital-to-analog converter) in which, the coarse value is encoded in 3-bits and the fine value is encoded in 8-bits. These currents are used to set the properties of the hardware circuits using the network.

### Mean-field network analysis

We reduced the network architecture to a minimal mean-field model, similar to^62^, to analyse its behaviour. The stability analysis of the coupled excitatory and inhibitory populations was done in order to choose the parameter set leading to the biologically plausible network behaviour. To be specific, we emulated the neural population bursts in response to transient stimuli recorded in the auditory cortex^26–28^ by producing the oscillatory behaviour near the fixed point of the excitatory-inhibitory network. The full mean-field network behaviours under five different protocols were then analyzed to conclude the consistency of the model prediction with the experimental observations. A necessary condition for true deviance detection generation in the model is given in the end.

The network is composed of multiple interconnected cortical columns. The thalamocortical inputs to each column linearly decrease along with the distance between the frequency of the stimulating tone and the column’s preferred frequency on the frequency axis. To be specific, every column is modelled by homogeneous excitatory and inhibitory populations that receive cross-adapted inputs from the adaptive layer upon thalamocortical stimulation, as shown in Fig. 1b.

#### Transient dynamics analysis

We first consider a mean-field rate version of two recurrently connected populations of excitatory and inhibitory neurons, receiving input from another population of adaptive neurons.

The dynamics of the adaptive population is defined by the following differential equations^59^:

1 $$\begin{aligned} \left\{ \begin{array}{l} \tau \dot{h}_a=-h_a+I_{ext} \\ \tau _a\dot{a}=-a+cA_a \\ \end{array}\right. \end{aligned}$$

where *a* represents the amount of adaptation accumulated by neurons, and $A_a$ is the mean population firing rate defined by a threshold-linear activation function of the mean synaptic input $h_a$:

2 $$\begin{aligned} A_a=[h_a-a]_+=\text {max}\left( h_a-a,0\right) \end{aligned}$$

Supposing that the synaptic current kinetics is much faster than the adaptation kinetics, namely, $\tau \ll \tau _a$, then Eq. (1) can be reduced to an one dimension equation by treating the synaptic current $h_a$ as instantaneously reaching its asymptotic value $I_{ext}$:

3 $$\begin{aligned} \tau _a\dot{a}=-a+c(I_{ext}-a) \end{aligned}$$

The solution for zero initial state $a(0) = 0$ is:

4 $$\begin{aligned} a(t)=\frac{c}{1+c}I_{ext}\left( 1-e^{-\frac{1+c}{\tau _a}t}\right) \end{aligned}$$

This means that if the external input $I_{ext}$ never stopped, the adaptive threshold *a* would exponentially approach to its asymptotic value $cI_{ext}/(1+c)$ with time constant $\tau _a/(1+c)$ and exponentially decay to 0 with time constant $\tau$ when later external input is removed (Supplementary data S1a, top). Correspondingly, since $A_a(t)=I_{ext}-a(t)$ when external input is on, starting from $I_{ext}$, the firing rate $A_a$ will exponentially decay to its asymptotic value $I_{ext}/(1+c)$ with $\tau _a/(1+c)$. $A_a$ will instantaneously drop to zero with no external input (Fig. 1c).

In addition, the dynamics of interacting excitatory and inhibitory populations is described as:

5 $$\begin{aligned} \left\{ \begin{array}{l} \tau _e\dot{h}_e=-h_e+w_{ei}A_i+w_{ee}A_e+w_aA_a \\ \tau _i\dot{h}_i=-h_i+w_{ie}A_e+w_{ii}A_i \\ \end{array}\right. \end{aligned}$$

Similarly, the mean firing rates of excitatory or inhibitory population $A_e$ or $A_i$ are defined as the rectified linear activation function of its mean synaptic inputs $A_e = [h_e]_+$ or $A_i = [h_i]_+$. $w_{ei}$, $w_{ee}$, $w_{ie}$, $w_{ii}$ and $w_a$ are the inhibitory-to-excitatory, excitatory-to-excitatory, excitatory-to-inhibitory, inhibitory-to-inhibitory and adaptive-to-excitatory connections, respectively.

To analyze the stability of the fixed point of the excitatory-inhibitory network, we rewrote the system (5) in the standard form by assuming the synaptic currents $h_e$ and $h_i$ are non-negative:

6 $$\begin{aligned} \left\{ \begin{array}{l}{\dot{h}_e=G_{1}(h_e, h_i)=\left[ ( w_{ee}-1)h_e+ w_{ei}h_i+w_aA_a\right] / \tau _e} \\ {\dot{h}_i=G_{2}(h_e, h_i)=\left[ ( w_{ii}-1)h_i+ w_{ie}h_e\right] / \tau _i }\end{array}\right. \end{aligned}$$

By definition, the Jacobian matrix *J* of above system at the fixed point $(h_{i0},h_{e0})$ is:

7 $$\begin{aligned} J\left( h_{i0}, h_{e0}\right) =\left. \left( \begin{array}{cc}{\partial G_{1} / \partial h_e} &{} {\partial G_{1} / \partial h_i} \\ {\partial G_{2} / \partial h_e} &{} {\partial G_{2} / \partial h_i}\end{array}\right) \right| _{\left( h_{i0}, h_{e0}\right) }= \left( \begin{array}{cc} {\left( w_{\mathrm {ee}}-1\right) / \tau _{\mathrm {e}}} &{} { w_{\mathrm {ei}} / \tau _{\mathrm {e}}} \\ { w_{\mathrm {ie}} / \tau _{\mathrm {i}}} &{} {\left( w_{\mathrm {ii}}-1\right) / \tau _{\mathrm {i}}} \end{array}\right) \end{aligned}$$

which is independent of the values of fixed point and external input $w_aA_a$. The stability of the fixed point is determined by the sign of the real parts of the eigenvalues of its Jacobian matrix *J*. The pair of eigenvalues $\lambda _{1,2}$ is given by:

8 $$\begin{aligned} \lambda _{1,2}=\frac{1}{2}\left( \frac{ w_{\mathrm {ee}}-1}{\tau _{\mathrm {e}}}+\frac{ w_{\mathrm {ii}}-1}{\tau _{\mathrm {i}}} \pm \sqrt{\left( \frac{ w_{\mathrm {ee}}-1}{\tau _{\mathrm {e}}}-\frac{ w_{\mathrm {ii}}-1}{\tau _{\mathrm {i}}}\right) ^{2}+\frac{4w_{\mathrm {ei}} w_{\mathrm {ie}}}{\tau _{\mathrm {e}} \tau _{\mathrm {i}}}}\right) \end{aligned}$$

Assuming that $\tau _{e}=\tau _{i}={\hat{\tau }}$, the above expression can be further simplified as:

9 $$\begin{aligned} \lambda _{1,2}=\frac{1}{2{\hat{\tau }}}\left( w_{\mathrm {ee}}+ w_{\mathrm {ii}}-2 \pm \sqrt{\left( w_{\mathrm {ee}}- w_{\mathrm {ii}}\right) ^{2}+4w_{\mathrm {ei}} w_{\mathrm {ie}}} \right) \end{aligned}$$

It turns out that the eigenvalues are a complex conjugate pair with positive real parts following the optimal parameter values given in Table 2. Therefore, the equilibrium $(h_{i0},h_{e0})$ is an unstable focus, which means that the phase-plane trajectory spirals away from the equilibrium until it joins a stable limit cycle induced by the rectification nonlinearity. The corresponding behaviours of $h_{e}$ and $h_{i}$ settle into periodic oscillations.

#### SSA in recurrent network with spike-frequency adapted input

The whole network of cortical columns is described by the following rate equations:

10 $$\begin{aligned} \tau \dot{h}_a^Q= & {} -h_a^Q+ \sum _{f=1}^{M}A\xi _fT_f^Q \end{aligned}$$

11 $$\begin{aligned} \tau _a\dot{a}^Q= & {} -a^Q+c[h_a^Q-a^Q]_+ \end{aligned}$$

12 $$\begin{aligned} \tau _e\dot{h}_e^Q= & {} -h_e^Q + \sum _{R=-1}^{1}w_{ee}^{|R|}[ h_e^{Q+R}]_+ + w_{ei}[ h_i^{Q}]_+ + w_a[h_a^Q-a^Q]_+ \end{aligned}$$

13 $$\begin{aligned} \tau _i\dot{h}_i^Q= & {} -h_i^Q + w_{ie}[ h_e^{Q}]_+ + w_{ii}[ h_i^{Q}]_+,\ Q=1,2,\ldots ,M \end{aligned}$$

where Eqs. (10) and (11) describe the network's dynamics of adaptive populations, which are expanded from single adaptive population dynamics defined by Eq. (1). Similarly, Eqs. (12) and (13) are adapted from Eq. (5), respectively representing the dynamical behaviour of excitatory and inhibitory populations within interconnected columns.

Every column *Q* is tonotopically organized along the frequency channel axis ordered from 1 to *M* by its preferred frequency $f=Q$ (best frequency). The temporal profile of external stimulation to a certain frequency channel *f* is given by the firing rate $A\xi _f(t)$, where *A* and $\xi _{f}(t)$ respectively denote the maximum amplitude and normalized temporal envelope (a fraction between 0 and 1) of the stimulus. In the simulation, $\xi _{f}(t)$ is chosen as a trapezoid pulse (50 ms duration with 5 ms onset/offset linear ramps).

$T_f^Q$ represents the relative strength of input column *Q* receives from frequency channel *f* compared with its best frequency channel *Q*. The values of $T_Q^f$ over all frequency channels form the tuning curve of column *Q*’s thalamic inputs. Here we choose a linear tuning curve:

14 $$\begin{aligned} T_f^Q = \left[ 1-\frac{|Q-f|}{\lambda } \right] _+ \end{aligned}$$

where $\lambda$ is the width of the tuning curve describing the level of cross-frequency thalamic inputs that cortical column *Q* receives.

The following demonstrates analytically the conditions at which the network is able to produce true deviance detection, i.e. response to deviant in the oddball protocol is larger than to the same deviant in ’Dev among std’. Taking inspiration from^14^, we define the accumulated adaptation load of column *Q* during certain protocol as:

15 $$\begin{aligned} L(Q)=\sum _{f=1}^{M} p_{f} T_f^{Q} \end{aligned}$$

where $p_{f}$ is the probability of appearance of the tone of frequency *f*. Here $T_f^{Q}$ can be considered as the extent of cross-frequency adaptation in column *Q* caused by tone stimulation of frequency *f*.

According to the network structure, the excitatory population in the recording column is excited by feedforward adapted inputs and lateral inputs from its neighbouring columns. The steady state of excitatory synaptic inputs to the excitatory population, in the recording column *N* and on the presentation of tones of frequency $N+1$ in a protocol is formulated as:

16 $$\begin{aligned} I_{Protocol}(N)= w_a C T_{N+1}^N B^{L(N)}+w_{ee}^1 f \left( w_a C B^{L(N+1)} \right) \end{aligned}$$

where $B < 1$ represents the steady-state response of adaptive population in a column to tones of the column’s best frequency within a sequence consisting of only tones of that frequency, normalized by the unadapted response *C*. Thus, the more adaptation loads the adaptive population has, the weaker its responses are, and accordingly, the lower inputs to the excitatory population. $f(\cdot )$ denotes the equivalent activation function of the excitatory population mediated by the inhibitory population, which is a monotonically increasing function given the parameter values.

In all simulation protocols, $N = 3$ and $\lambda = 2$. Hence, in “Standard in OD” protocol, $L(3)=0.25T_2^3+0.75T_4^3=0.5$ and $L(4)=0.25T_2^4+0.75T_4^4=0.75$. Similarly, $L(3)=0.5T_2^3+0.5T_4^3=0.5$ and $L(4)=0.5T_2^4+0.5T_4^4=0.5$ for “Equal”; $L(3)=0.75T_2^3+0.25T_4^3=0.5$ and $L(4)=0.75T_2^4+0.25T_4^4=0.25$ for “Deviant in OD”; $L(3)=0.25T_4^3=0.125$ and $L(4)=0.25T_4^4=0.25$ for “Dev alone” and at last $L(3)=0.25T_1^3+0.25T_2^3+0.25T_4^3+0.25T_5^3=0.25$ and $L(4)=0.25T_1^4+0.25T_2^4+0.25T_4^4+0.25T_5^4=0.375$ for “Dev among std”. Due to $B < 1$, we get $I_{Standard\ in\ OD}(3)< I_{Equal}(3)< I_{Deviant\ in\ OD}(3) < I_{Dev\ alone}(3)$.

In order to demonstrate TDD, we need to let $I_{Deviant\ in\ OD}(3) > I_{Dev\ among\ std}(3)$, that is to say, the following difference should be positive:

17 $$\begin{aligned} w_a C T_{N+1}^N \underbrace{\left( B^{0.5}-B^{0.25} \right) }_{<0} + w_{ee}^1\underbrace{\left[ f\left( w_a A B^{0.25}\right) - f\left( w_a A B^{0.375}\right) \right] }_{>0}>0 \end{aligned}$$

The first and second term in the expression is respectively negative and positive. Hence, possible ways of tuning parameter to achieve TDD are to make the second term more positive by increasing inter-columnar connection $w_{ee}^1$ and/or decreasing the intra-columnar adaptive-to-excitatory connection $w_a$.

All the model parameters are summerized in Table 2. The network was numerically simulated by Euler’s method with a time step $1\times 10^{-4}$ s.

**Table 2:** Parameters in the mean-field model.

Notation	Description	Optimal value	Operating range
*M*	Number of columns	5	
$\lambda$	Width of tuning curve	2	
$\tau _a$	Time constant for adaptation process	1 s	
$\tau$	Membrane time constant of adaptive population	$1\times 10^{-3}$ s	
$\tau _{e}$	Membrane time constant of excitatory population	$5\times 10^{-3}$ s	
$\tau _{i}$	Membrane time constant of inhibitory population	$5\times 10^{-3}$ s	
$w_{ee}^0$	Intra-columnar excitatory-to-excitatory connection weight	3.25	[3.2, 3.5]
$w_{ee}^1$	Inter-columnar excitatory-to-excitatory connection weight	0.2	[0.15, 0.35]
$w_{ie}$	Intra-columnar excitatory-to-inhibitory connection weight	1.8	[1.75, 1.95]
$w_{ei}$	Intra-columnar inhibitory-to-inhibitory connection weight	-3	[-3.3, -2.85]
$w_{ii}$	Intra-columnar inhibitory-to-inhibitory connection weight	-1	[-1.1, -0.7]
$w_{a}$	Connection weight between adaptive and excitatory populations	0.5	(0, 2.5]
*c*	A constant to scale the rate of adaptive population	20	(1, 100]

Steps of 0.05, 0.1 and 1 are used to linearly search the operating ranges of the excitatory and inhibitory connection, adaptation connection weights and adaptation scaling factor, respectively. The parameter value is valid if the CSI metrics for SSA and TDD of the model based on that parameter value are positive. Values exceeding 100 for the adaptation scaling factor have not been tested since SSA and TDD vary very slightly when increasing the parameter linearly. The setup of simulated protocols is the same as that in Fig. 1.

### Neuromorphic implementation

#### Neuromorphic hardware

The network was implemented on the Dynamic Neuromorphic Asynchronous Processor (DYNAP-SE) chip^18^ which allows the implementation of reconfigurable and real-time neural networks, based on arrays of silicon neurons and synapses. The DYNAP-SE is a fully asynchronous mixed-signal spiking neural network processor (Fig. 3c). It is composed of analog spiking neurons that emulate the biophysics of their biological counterparts and neuromorphic circuits which can emulate the synaptic dynamics in real-time^19^. It comprises 1000 adaptive exponential integrate-and-fire neurons with four different synapse types per neuron. Each neuron has a content addressable memory block, containing up to 64 addresses representing the presynaptic neurons that the neuron can be connected to. This limited number of incoming connections cannot be changed and largely defines how we set the exact number of connections per neuron. Asynchronous digital peripheral input/output logic circuits are used to receive and transmit spikes. The analog circuit parameters dictating the behaviour and dynamics of the neurons and synapses are set by an on-chip programmable bias generator. Rounds of parameter sweeps in the initial setup of the hardware platform were performed to find a suitable initial condition and operating point of the circuits (See Supplementary data S4 for an example of spike activity of the silicon neurons on the DYNAP-SE board and Supplementary data S5 for the details of the initial steps).

#### Stimulus presentation protocol

Commonly, two stimuli that evoke similar responses in the recording site are used in an oddball (OD) paradigm^60^ and are presented repetitively and randomly within an OD, one (the standard stimulus) with a high probability of appearance and the other one (the deviant stimulus) with a lower probability of appearance. Stimuli were represented as input spikes that were sent to the network by an external spike generator. Stimuli were usually presented with a duration of 100 ms with an interstimulus interval (ISI) of 1 s (onset-to-onset) as in the study of Ulanovsky et al. (2003)^60^in which a range of ISI (from 375 to 4000 ms) were tested. Each stimulus was presented with a constant frequency f. Sequences were usually composed of 100 stimuli as the behaviour of the model in response to a sequence of stimuli was the key interest in this paper. The number of stimuli was determined by previous work on SSA^14,16^. The analysis of SSA usually uses a “traditional” OD (Fig. 2a) consisting of the use of two stimuli with two frequencies (f1 and f2) with different probabilities of appearance: the “deviant” had a probability of P = 25% while the “standard” had a probability of 1- P = 75%. These two stimuli stimulated two separate columns in our network ( “deviant” and “standard” columns respectively). In this paradigm, repeated presentation of the standard stimulus interspersed with rare and random deviant stimulus presentation was used and the measurement of SSA was made by comparing the neuronal response of our network obtained when the deviant was presented to the one when the standard was presented. To study the existence of true deviance detection, additional control OD paradigms were used. The “Equal” protocol in which both stimuli were presented randomly to the network with a probability of appearance of 50% to make sure that there was no preferential behavior from the stimuli. The Deviant alone protocol, in which the deviant was presented to the network as the same probability as in the “traditional” OD and all standard presentations were replaced by silence. The response from this protocol was used to normalize our data in Fig. 2h as it provided the largest response of our network. The “Deviant among Standards” protocol (“Dev among std”) consisted of a sequence of four equiprobable (25%) stimuli, including the two stimuli used in the “traditional” oddball condition and each column of the network was stimulated in a random manner. In these trials, there was no violation of expectations, contrary to the “traditional” OD in which the deviant infringe the regularity set by the repetitive standard presentation.

#### Data analysis

The readout for spike count was taken from the middle column (column 3) of the network. For all plots, the MFR was calculated as the average firing rate of all the neurons in the population over windows of 100 ms, starting at stimulus onset and ending 10 ms after stimulus offset. When existing, 2–3 overactive noisy neurons (i.e., neurons continuously firing in the absence of input spikes) were removed from the analysis. The initial stimulation as well as the stimulation by the rarely occurring deviant stimulus will lead to the activation of the targeted column driven by intracolumnal recurrent connections. After several presentations of the same stimulus (i.e. the “standard” stimulus), the feedforward input coming from the adaptive layer will become more adapted and therefore lead to a lesser activated column. SSA is usually quantified by an index (common SSA index, CSI) which defines the sensitivity of neurons to stimuli when they are presented as deviant stimuli compared to when they are presented as standard stimuli^11,60^. This index is obtained following the “traditional” OD paradigm (detailed in section Different OD paradigms and behaviour of our network) in which the network is presented with two stimuli, one with a probability of appearance of 75% and the other with a probability of 25%. CSI, defined as follows, is then computed:

$$\begin{aligned} CSI = \frac{d(f1) + d(f2) -s(f1) -s(f2)}{d(f1) + d(f2) + s(f1) + s(f2)} \end{aligned}$$

where *d*(*f*) and *s*(*f*) are the responses measured as spike count to frequencies *f*1 or *f*2 used as either the deviant (*d*) or the standard (*s*) stimulus. CSI value ranges between -1 and +1 with more positive values reflecting a greater response to the deviant stimuli and therefore, a stronger SSA response. At first, the whole CSI was computed for the entire oddball paradigm and then the overactive noisy neurons (i.e., neurons firing in the absence of any stimulus, which results from the intrinsic properties of the neuromorphic board such as noise and in-built mismatch) were removed from the analysis. The latter was performed on ten different runs for 100 stimulation presentations.

To analyse true deviance detection, the above-mentioned CSI equation was modified^63^ accordingly and instead of using the deviant and standard responses, we used the deviant response to the “Deviant in OD” and to the “Deviant among Standards” protocols:

$$\begin{aligned} CSI (true\ deviance) = \frac{Deviant\ in\ OD\ (Dev\ Response) - Dev\ among\ std (Dev\ Response)}{Deviant\ in\ OD\ (Dev\ Response) + Dev\ among\ std (Dev\ Response)} \end{aligned}$$

If the CSI (true deviance) is superior to 0 then the model displays true deviance detection in the “Deviant in OD” when compared to the response from the deviant response in the “Deviant among Standards”.

## Supplementary Information

Supplementary Information.

## Data Availability

The data and code used for the neuromorphic experiments are provided on the code hosting platform github in: https://github.com/NatINI/NVS_et_al_2021_SSA. The code for the mean-field model can be accessed via https://github.com/ChaoHan-UoS/AuditorySSA_Hardware.
