# Supplementary material for: A robust model of Stimulus-Specific Adaptation validated on neuromorphic hardware
Source: Sci Rep. 2021 Sep 9;11:17904. doi: 10.1038/s41598-021-97217-3 (PMC8429557; doi:10.1038/s41598-021-97217-3)
Supplement: Supplementary file 1 — Supplementary Information. [file 41598_2021_97217_MOESM1_ESM.pdf]

Supplementary material for the manuscript: A robust model of Stimulus-Specific Adaptation validated on neuromorphic hardware

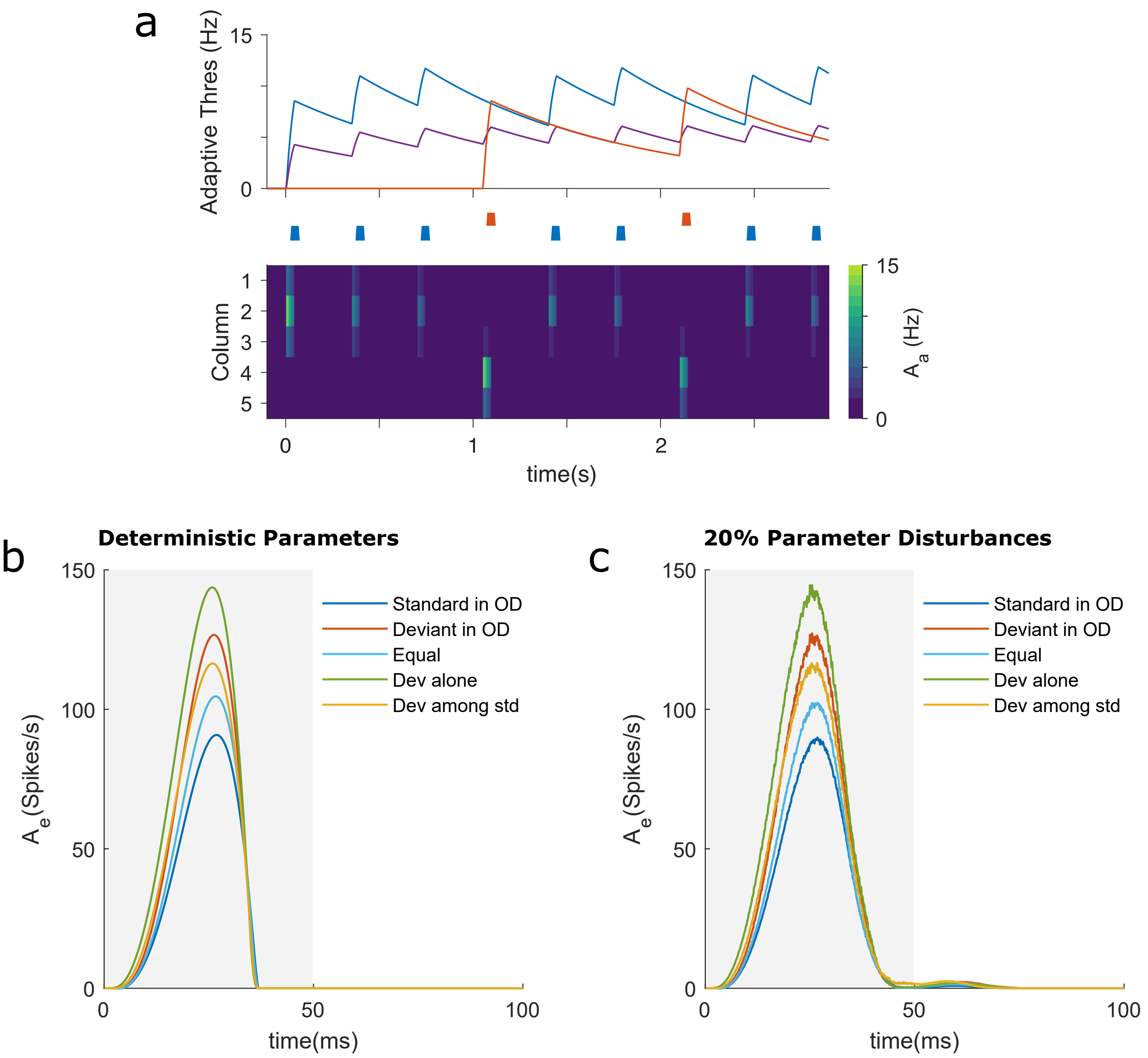

Figure S1. Activities across columns in adaptive layer and average responses of recording column in different protocols. (a) The amounts of adaptation or adaptive threshold a accumulate immediately upon the presentation of stimuli and gradually release during the inter-stimulus interval. Adaptation in the standard column (blue) is often more substantial than the deviant column (red). The same amount of adaptation is induced in the recording column (purple) independent of the identity of stimuli due to the symmetry of the spatial tuning curve of thalamocortical input (top). Stimuli sequence is displayed under the plot. Accordingly, the adaptive population activities A_a in the standard column (column 2) is often weaker than the deviant (column 4) whereas same extent of activities is induced in the middle column (column 3) after the first few stimuli (bottom). **(b)** A tone stimulus is presented in different conditions as deviant (red, probability of occurrence of 25%), standard (blue, probability of occurrence of 75%), one out of two stimuli with equal probability (cyan, probability of occurrence of 50%), deviant among many standards (yellow, 25% probability of occurrence for each of 4 frequencies) or deviant alone against silence (green, probability of occurrence of 25%). The gray shadow marks the duration of stimulus. Data are averaged over each protocol presented with 800 stimuli. **(c)** Analogous to **(b)**, but 20% real-time parameter disturbances are introduced.

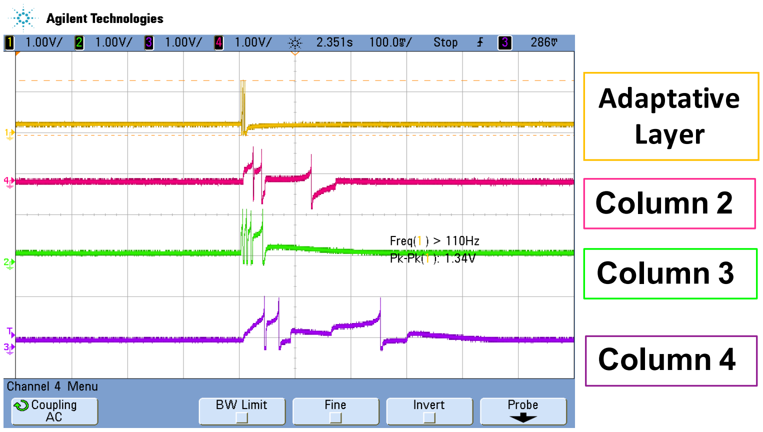

Figure S2. **PS propagation through the network**. These oscilloscope traces demonstrate the propagation of spikes from the column where spikes were sent to the two adjacent columns. As previously reported in SSA in vivo study a couple of milliseconds are necessary in order for the spikes to reach the next column from the column where the stimulation was applied

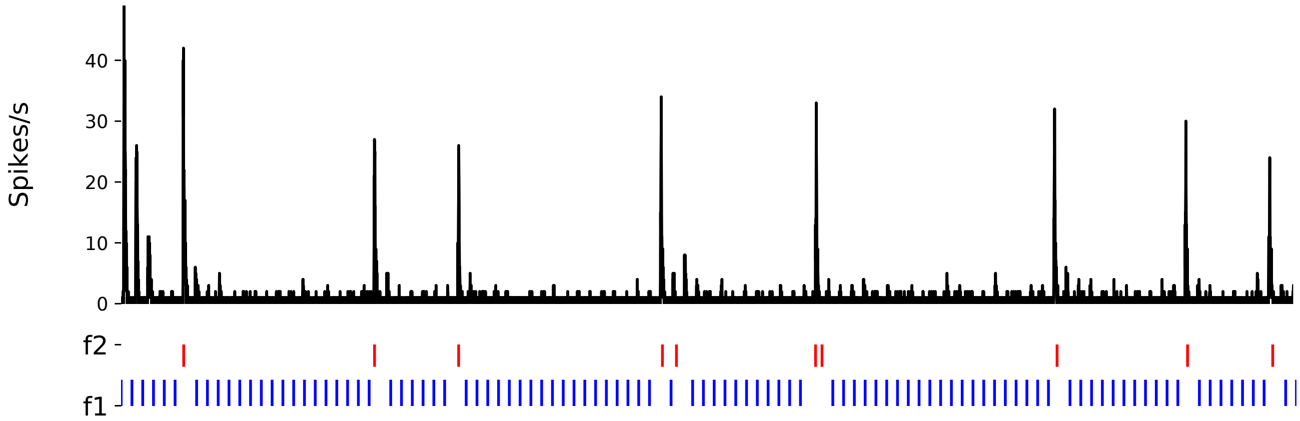

Figure S3. **Adaptation occurring in the neuromorphic hardware**. This figure shows how neurons require a minimum of 3 stimulation to get fully adapted. SSA occurs when the deviant is presented (red label under the plot) and does not when the standard is presented (blue label under the plot) as the column is already adapted. In all OD paradigm stimulus duration = 100 ms and inter-stimulus interval (ISI) = 1 second.

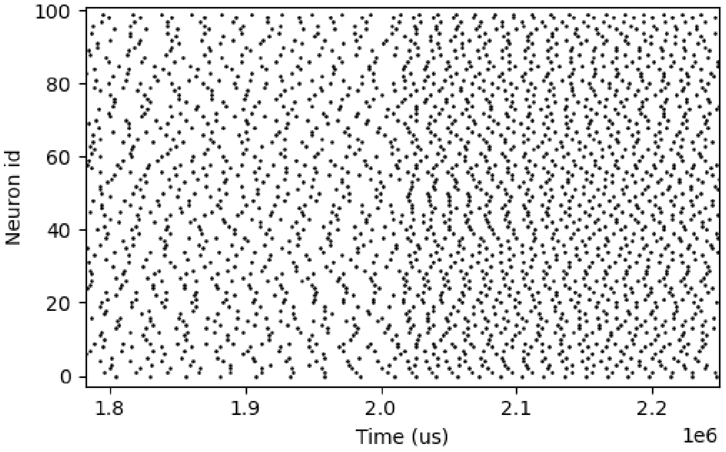

Figure S4. Example of rasterplot for the spike activity of the silicon neurons on the neuromorphic chip. Here the DC current injection parameter is set to produce an average firing rate of 40Hz. After 2 seconds, we produce a step change in the input DC current parameter, to increase the average firing rates to 65Hz. As shown, there are no visible ringing or oscillatory behaviors and the neurons reach their steady-state output with no instability.

Supplementary data S5. Initial tuning steps for the neuromorphic hardware. To implement a recurrent neural network inspired by the work of[1] into our neuromorphic hardware, we initially checked if the synaptic and neuronal parameters of the hardware (such as time constants, efficacy, or refractory period) were at a suitable initial condition and operating point. We tuned in the following sequence 1) the neuron's parameter, 2) the synapse's parameters and 3) set the weights. We switched off first all synapses (so that they do not leak into the neurons), then the spike frequency adaptation, and finally all neurons.
1) For the neuron's parameters, we looked first at the neuron's time constant bias (Tau_N, generally around 40ms) value that will be about 10 times for the slow synapse Tau (NMDA synapses) or 1/4 for the fast synapse Tau (AMPA synapses). To find the Tau_N, we worked with DC steps to see how fast the neuron was able to recover from a spike. We applied a subthreshold current to the neuron for about 0.5s making sure at the same time that the neuron was not spiking. We looked at the decay of the membrane potential (V_{mem}) in an oscilloscope and the Tau_N was roughly 2/3 of the time it takes for the V_{mem} to decay. Second, we searched for the Neuron threshold bias (Thr_N) which was 2 to 3 times the value of Tau_N. Third, we looked at the neuron refractory period bias, for which we had to inject a large DC current to make the neuron fire at a very high firing rate. We set the refractory period so that the maximum firing rate was between 200Hz and 300Hz.
2) For the synapses parameters, we determined the synapses' time constant bias (NPDPI_Tau). TAU determines the strength of the leak and small values correspond to a long synaptic time constant. To find the synapses' time constant, we set the Tau_N of the tested core to a high value so that the neuron directly follows the synaptic input. We used the spike generator to apply input spikes through the synapses to the neuron and look at the decay of the V_{mem} in oscilloscope. The neuron's V_{mem} directly followed the synapse now. Hence, the synapses' time constant was roughly 2/3 of the time it takes for V_{mem} to decay. For the synapses threshold bias (NPDPI_Thr), we set it up to 2, or at most 3, times the NPDPI_Tau values found. The goal is that there is no spontaneous firing of neurons anymore, while still keeping the Taus low. If some neurons were still firing spontaneously, they could be silenced by assigning them the second Tau bias instead of the first one and setting this bias very high.
3) For establishing the weight of the synapses (PS_Weight) we monitored the spike response of single neurons for different synapse types. We started with slow excitatory synapses and send about 100Hz-1kHz input spikes and balanced the weight and the pulse width. Pulse width is global for all synapse types, so we set it once and then adapted the biases for each synapse type. Weights for fast synapses were usually about 10 times smaller than for slow ones. For the inhibitory synapses, we also send input via an excitatory synapse and observe how the inhibitory input affects the neuron response.
We at the end set up our network, observe its activities and apply fine-tuning when necessary. We ran the series of experiments from figure 2 to check the response from the adaptive layer (figure 2a) and the column (figure 2b). We then analyzed the response of the network to different input stimuli to check the existence of a threshold (figure 2c), and finally the existence of the Populations Spikes propagation through the network (figure 2d). After this series of control experiments, we ran different oddball paradigm tests ("traditional" and controls OD) and investigated the neuronal response of our network to see its potential in producing SSA (figure 2e-h). This approach was iterated systematically, to find the optimal parameters for the network.

References

1. Yarden, T. S. & Nelken, I. Stimulus-specific adaptation in a recurrent network model of primary auditory cortex. *PLoS computational biology* **13**, e1005437 (2017).
